# The standardized surgical approach improves outcome of gallbladder cancer

**DOI:** 10.1186/1477-7819-5-55

**Published:** 2007-05-21

**Authors:** Stefan Scheingraber, Christoph Justinger, Tatiana Stremovskaia, Malte Weinrich, Dorian Igna, Martin K Schilling

**Affiliations:** 1 Department of General-, Visceral-, Vascular- and Paediatric Surgery, University Hospital, University of the Saarland, D-66421 Homburg, Germany; 2 Institute of Pathology, University Hospital, University of the Saarland, D-66421 Homburg, Germany

## Abstract

**Background:**

The objective of this study was to examine the extent of surgical procedures, pathological findings, complications and outcome of patients treated in the last 12 years for gallbladder cancer.

**Methods:**

The impact of a standardized more aggressive approach compared with historical controls of our center with an individual approach was examined. Of 53 patients, 21 underwent resection for cure and 32 for palliation.

**Results:**

Overall hospital mortality was 9% and procedure related mortality was 4%. The standardized approach in UICC stage IIa, IIb and III led to a significantly improved outcome compared to patients with an individual approach (Median survival: 14 vs. 7 months, mean+/-SEM: 26+/-7 vs. 17+/-5 months, p = 0.014). The main differences between the standardized and the individual approach were anatomical vs. atypical liver resection, performance of systematic lymph dissection of the hepaticoduodenal ligament and the resection of the common bile duct.

**Conclusion:**

Anatomical liver resection, proof for bile duct infiltration and, in case of tumor invasion, radical resection and lymph dissection of the hepaticoduodenal ligament are essential to improve outcome of locally advanced gallbladder cancer.

## Background

In the recent surgical literature therapy of gallbladder cancer (GC), which has traditionally been viewed with therapeutic nihilism, has documented an increase of 5 year survival rates from 5–12% up to 38% [1]. Because the survival of patients treated by palliative chemotherapy or radiation is poor, limited to months, an aggressive approach to locally confined disease is justified. However, there is considerable controversy what exactly constitutes that "aggressive surgical approach" [2]. The armamentarium of surgical procedures mainly comprised liver resection, common bile duct resection, lymph node dissection in the hepaticoduodenal ligament and -especially practiced in Japan- concomitant pancreatoduodenectomy or lymph dissection of the interaortocaval compartment. With respect to the liver resection, the variety of procedures ranged from non-anatomical wedge resections, to anatomical parenchyma sparing segment IVb/V resections up to extended right or left hemi-hepatectomies. The indications for several procedures depend more on center specific algorithms than on randomized trials. In our department there was a switch in March 2001 to a more aggressive surgical approach in the treatment of gallbladder cancer with a standardized approach comprising anatomical segment IV (round ligament approach resection)/segment V resection, systematic lymph dissection of the hepaticoduodenal ligament (HL) and resection of the common bile duct (CBD) to reach tumor free margins. Before March 2001, the surgical approach to gallbladder cancer was largely dependent on the individual surgeon's decision. The aim of this study was to examine the extent of resection, the complication rate, the intraoperative and microscopic findings and at least survival rates of patients treated in the time period of the last 12 years with special respect to the impact of the standardized approach on outcome.

## Patients and methods

Patients seen at the Department of General Surgery of the University Hospital of the Saarland from November 1994 to January 2006 with the histologically proven diagnosis of GC were identified from the national tumor registry, the admission diagnosis and surgical procedures data bank. Data were collected retrospectively from chart review including operation reports and histological examination and survival time was calculated after telephone interviews with patients or practitioners of the patients. All patients with a curative or a palliative surgical intention were included, as well as patients reoperated for recurrent GC.

**Figure 1:**
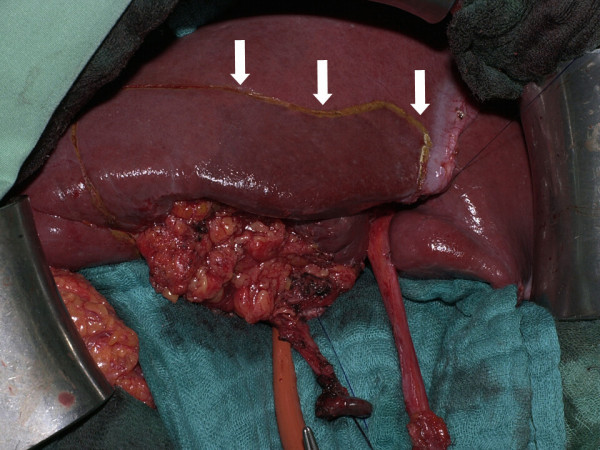
Gallbladder cancer infiltrating the liver. En bloc resection of the tumor mass with liver segments IVb and V. Arrows demonstrate demarcation of segment IVb after round ligament approach.

**Figure 2:**
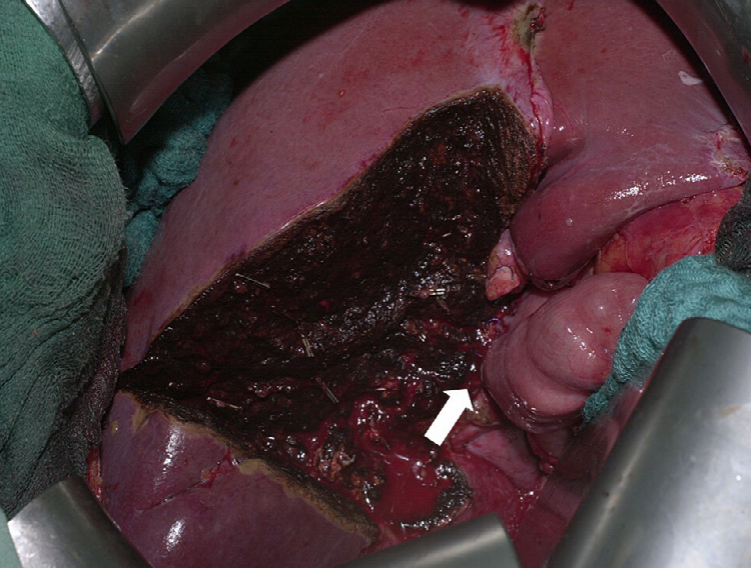
Well perfused liver after resection of segments IVb/V, with high intrahilar biliary-intestinal anastomosis (arrow).

After median laparotomy and exclusion of distant metastases (e.g. liver metastases or peritoneal seeding) cholecystectomy (CHE) with frozen section of the macroscopically unsuspicious cystic duct was performed. Whenever tumor infiltration in the gallbladder bed of the liver or the CBD was suspected from preoperative radiological examinations or the intraoperative macroscopic aspect we intended to perform an *en bloc *resection of the gallbladder together with segment IVb/V resection or with CBD resection. In cases of previous CHE the cystic duct was identified and a frozen section was also examined from the resection margin. The next step was the incision of the lesser omentum followed by a lymph dissection from the left gastric artery to the celiac trunk as well as lymph dissection from the common hepatic artery until the branches of the right and left hepatic artery (LN 7, 8a, 8p, 9, 12). Then the lymph nodes between the portal vein and the CBD were resected (LN 12p, 13a). In case of bile duct infiltration the CBD was transected just above the pancreatic border and was resected en bloc with the lymphatic tissue of the HL and the dorsopancreatic lymph nodes after the Köcher's procedure until the right border of the aorta (LN 13 b). After that the bifurcation of the hepatic duct was mobilized by lowering the hilar plate. From the hilar transaction of the common hepatic bile duct also a frozen section was performed to prove for tumor free resection margins. After the transaction of the bile duct and the lymph dissection liver resection of the gallbladder bed (Couinaud segments IVb and V) was done using the round ligament approach and selective clamping of the anterior pedicle of the right portal vein. After devascularization of the vessels the liver bed became demarcations (figure 1) and the liver parenchyma was resected anatomically along this margin with the use of an ultrasound dissector as well as vascular clips and PDS 4/0 sutures of major vessels and bile ducts (figure 2). Blood coagulation was additionally achieved by argon beamer coagulation and the use of tachosil^®^. After common hepatic bile duct resection hepaticojejunostomy was performed with a transmesocolic lifted jejunal loop. Intestinal continuity was achieved with a *Roux en Y *side-to-end anastomosis. When the tumors infiltrated neighboring organs (especially the right colonic flexure) an *en bloc *resection was performed to achieve complete histological removal of the tumor at the resection margins (R0). In cases of previous laparoscopic CHE excision of the port site where the gallbladder was removed was done at the end of the procedure.

As no lymph node metastases and a good survival were reported in T1a tumors [3] simple CHE was considered as adequate radical for this group. In contrast, in patients with T1b tumors, we perform extended resections according to the findings of the French Surgical Association Survey [4].

The survival rates were estimated and plots constructed by the Kaplan-Meier method with the aid of a statistic software (SigmaStat 3.0^®^, SPSS). Tumors were staged according the TNM-classification and the actual classification of the International Union Against Cancer (UICC) [5]. Differences among the survival rates were compared with the log-rank test. A p-value of less than 0.05 was considered significant. Numerical data were expressed as mean ± standard deviation for normal distributed and as median with range for non-normal distributed values. Differences of mean values were compared with the student t-test and for median values with the Mann-Whitney-U-test.

## Results

### Demographics

Fifty three patients with GC were identified. Thirty nine were female and fifteen male, with an average age at first presentation of 67 (39–88) years. Median hospital stay was 15 (3–77) days. Twenty one patients were operated with a curative intention. Of these seventeen patients had undergone previous CHE and were reoperated. In the remaining patients there was only a palliative procedure possible due to advanced tumor stages with distant metastases, recurrent disease or because of significant medical co morbidity. About half of the patients in palliative therapy group underwent staging laparotomy (n = 14) or laparoscopy (n = 1). In eight patients reoperation due to recurrent disease was performed. Even when tumor resection was performed in this group, the operation was considered as palliative. In the remaining patients varying palliative procedures were performed ranging from cyrotherapy of liver metastases to implantation of a venous port system for palliative chemotherapy. In three patients bypass surgery was done, two patients with gastroduodenal stenosis received gastroenterostomy and in one patient with colonic infiltration of the right flexure received ileotransversostomy.

### Disease stage

In Table 1 the UICC stages of the GCs and the percentage of R0-resections is shown. There was a large proportion of R0 resections after implementation of the standardized approach even in patients with lymph node metastasis and in tumors infiltrating two neighbored tissues. However, no curative resection was possible in stage IV patients and also the chance for curative resection in recurrent disease was quite low.

**Table 1:** Staging (UICC 2002 classification) and percentage of R0 resection of the gallbladder cancers

**Stage**	Number	Percentage of R0 resections
**I b**	9	100%
**II a**	2	100%
**II b**	9	77%
**III**	6	66%
**IV**	19	0%
**recurrent tumour**	8	12%

### Recurrent disease

During the study period of 12 years we treated 8 patients with recurrent disease of GC (Table 2). One half of the patients had undergone CHE in another hospital previously, the other half was treated in our department from the beginning. The most marked finding was that no dissection of the HL was performed in three fourths (6/8) of the patients, especially in the patient without completion operation after CHE with histological diagnosis of GC, who developed the tumor behind the HL. Another problem was that tumor free resection margins could not be achieved in one patient who developed early gastric stenosis. Half of the patients with recurrent disease underwent non-anatomical resection. However, tumor recurrence was also seen in four patients after anatomical segment IVb/V liver resection.

**Table 2:** Patients treated with recurrent disease after previous curative surgery for gallbladder cancer (n = 8).

**Gender/Age [years]**	**TNM**	**Procedure**	**Time until reintervention [months]**	**Problem**	**Palliative procedure**	**Oucome**
female,49	TxNxM0	non anatomical liver resection after previous open CHE, no lymph dissection, no exploration of CBD	22	icterus	hepaticojejunostomy	40 months later alive
male, 69	T2NxM0	non anatomical liver resection after previous open CHE, no lymph dissection, no exploration of CBD	6	liver metastases seg. VII, tumour recurrence seg. IV/V	bisegmentectomy seg. IVb/V, cryotherapy seg. VII	died 4 months later
female,69	T2N1M0	non anatomical liver resection Seg. IVb after previous laparoscopic CHE, radical lymph dissection, excision of port sites and tumour free resection margins CBD	12	icterus	seg. III-bypass, gastroenterostomy	died 3 months later
male, 72	T2NxM0	T2 GBC after open CHE, no completion operation	23	tumour between hepaticoduodenal lig. and pancreas	exploration	died 6 months later
female, 68	T3N1M0	anatomical liver resection Seg. IVb/V after open CHE, lymph dissection, no tumour free resection margin CBD	3	gastric stenosis, liver abscess	interventional abscess drainage, implantation venous port system	died 1 month later
male, 64	T3NxM0	anatomical liver resection Seg. IVb/V after open CHE, no lymph dissection, tumour free resection margin CBD	1	duodenal stenosis	gastroenterostomy	died 1 month later
female, 60	T3NxM0	bisegmentectomy seg. IVb/V en bloc with CHE, no lymph dissection tumour free resection margin of CBD,	11	skin metastasis	resection	died 13 months later
male, 74	T4NxM0	non-anatomical wedge resection of seg. IVb during CHE, no lymph dissection, no tumour free resection margin of CBD	2	tumour progress with liver infiltration	cryotherapy	lost for follow up

Abbreviations: CHE = cholecystectomy, CBD = common bile duct, seg. = Couinaud liver segment

### Operative procedures and histological findings

To examine the tumor spread in relation to the tumor state histological findings of all operated patients (curative as well as palliative) were analyzed (Table 3). As not in every case complete resection of liver segments IVb/V, lymph dissection of the HL and resection of CBD was performed, the proportion of histologically proved tumor infiltration in the resected specimens is documented in parentheses. Additionally, the incidence of metastases and involvement of lymph nodes at the celiac trunk indicating advanced lymphatic spread are shown. There was only one T1b tumor with segment IVb/V liver resection and lymph dissection of the HL and no evidence of liver and lymph node infiltration. In eleven of 17 patients with T2 tumors segment IVb/V liver resection was performed. By definition, there was no liver infiltration found in this group. However, in 25% of 13 resected lymphatic tissue specimens of the HL there were signs of lymphatic spread. Additionally there was an infiltration of the CBD in already 25% of T2 tumors. In this study there were 17 patients with T3 tumors. Four of these 17 (24%) patients had undergone open CHE previously and underwent the completion operation. In only half of the patients of the T3 group liver resection was performed, as there were already signs of distant tumor spread like positive lymph nodes at the celiac trunk or disseminated metastases found. Finally, in T4 patients there was a tremendous high incidence of distant or disseminated metastases and an obligatory infiltration of the HL. In patients with disseminated metastases liver resection was not indicated and therefore there were also three patients with metastases classified as Tx. In eight patients reoperated for completion operation after laparoscopic CHE the skin and soft tissue of the port site was excised (data not shown). However, in no specimen of port sites tumor cells were found by the pathologist.

**Table 3:** Operative procedures according to tumor staging (UICC 2002 classification).

**T-State**	**Liver Resection **(liver infiltration)	**Dissection of Hepatico-Duodenal Ligament **(lymph node infiltration)	**Biopsy coeliac trunc nodes **(lymph node infiltration)	**CBD resection/R0 cysticus **(CHDB infiltration)	**Metastases**
T1b n = 1	1/1 (0%)	1/1 (0%)			0%
T2 n = 17	11/17 (0%)	13/17 (25%)	5/17 (0%)	4/17 (25%)	0%
T3 n = 14	7/14 (100%)	7/14 (67%)	2/14 (50%)	5/14 (40%)	liver n = 1 peritoneal carcinosis n = 3
T4 n = 17	6/17 (100%)	4/17 (100%)	2/17 (100%)	0/17	liver n = 5, pancreas n = 1, lung n = 1, peritoneal carcinosis n = 6
Tx n = 4	0/4	0/4		0/4	liver n = 1, peritoneal carcinosis n = 2

Reported is first the number of patients who received the certain procedure and than the total number of patients with the certain tumor stage. In parentheses the percentage of histological proved tumor infiltration in the resection specimens is demonstrated.

In thirteen cases anatomical resection was performed, in one case right sided hemihepatectomy and in remaining 10 patients non-anatomical liver resection (Table 4). Mean intraoperative transfusion requirements of packed red blood cells was 0.6 ± 0.3 units in the anatomical and 1.1 ± 0.9 units in the non-anatomical group. Eight patients in the anatomical and eight patients in the non-anatomical group did not need any intraoperative blood transfusion. There was also only one patient in each group needing administration of 5 or 4 fresh frozen plasma intraoperatively. Time of the operation including lymph dissection of the HL was also not different between anatomical and non-anatomical liver resection.

**Table 4:** Demographic data for the Standardized and Individual approach in 21 patients treated with curative intention (R0 resections).

	**Individual Approach n = 9**	**Standardized Approach n = 12**
**Age**		
[Mean ± SE (Range)]	65 ± 12 (60–80)	71 ± 6 (40–78)
**UICC**		
1b	6	1
2a	0	2
2b	2	8
3	1	1
**Liver Resection**		
Anatomical	2 (22%)	12 (100%)
Non-anatomical	6 (67%)	
**Lymphdissection**		
Hepaticoduodenal Ligament	6 (67%)	12 (100%)
**CBD**		
Exploration/Resection*	1 (11%)	12 (100%)

In parentheses the percentage of the procedure in relation to the whole treatment group (n = 9 patients with individual and n = 12 patients with the standardized approach)

### Postoperative complications and mortality

A total of 30 of 53 patients had an uneventful postoperative course during their hospital stay. There were some minor complications like postoperative urinary tract infection in one patient, delayed wound healing in one patient, pleural effusion in 4 patients, pneumonia in 2 patients and prolonged intestinal atonia in 3 patients. In three cases there was an anastomotic leakage of the hepaticojejunostomy which made reoperation necessary. Two of these patients developed liver abscess. Five patients died during the hospital stay. Two deaths could be directly related to the surgical procedure in one patient with postoperative bleeding due to erosion of the hepatic artery after radical lymph dissection and in another patient who developed postoperative liver failure. Two other patients died due to medical reasons (cardiac failure and pulmonary embolism). Finally one patient died due to tumor progress already during her hospital stay after staging laparoscopy.

### Survival

**Figure 3:**
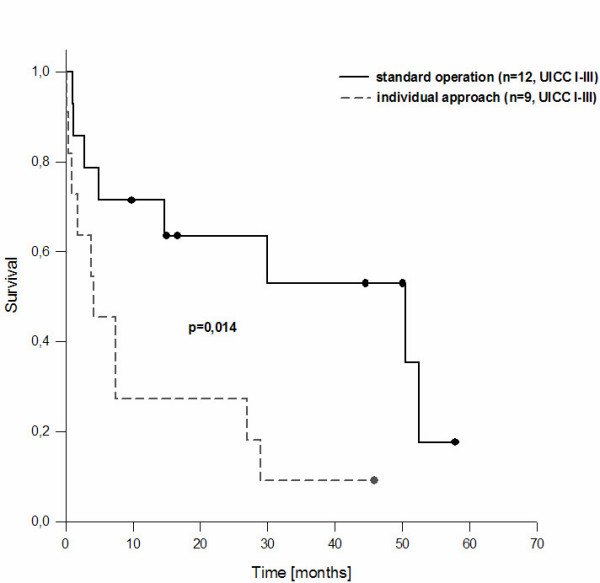
Survival of curative resection of gallbladder carcinoma following the standard operation and with an individual approach (formally RO resections, but no resection of all components of the standard operation) (p < 0.014 log rank test).

Median follow-up was 13 (0–51) months. To further clarify the role of the standardized approach on survival all 21 patients with T1, T2 and T3 tumors, operated with curative intention (R0 resections), were divided the patients in a group with standardized and individual resections (Table 4). Standardized resection was defined as liver resection, lymph dissection of the HL, exploration of the CBD and (in case of tumor infiltration) resection until tumor free resection margins are achieved. In the individual group one of these three components was not performed due to the surgeons' individual decision. Formally, the histological analyses of the resection specimens revealed R0 resection, although there was no unique information about the tumor infiltration of the gallbladder bed, the HL and the CBD. In most cases the deviation of the individual approach compared with the standard was that no lymph dissection of the HL was performed and no frozen section of the cystic duct was performed to exclude bile duct infiltration. Survival analysis of the patients treated according the standardized approach compared with patients treated according an individual approach showed significant better survival for patients undergoing standard resection with a median survival of 14 months compared to 7 months in individual resection group (Figure 3).

## Discussion

Until the last 10 years GC has always been associated with dismal prognosis, due to the asymptomatic growth of the tumor and finally the infiltration of the surrounding structures as the hepatic artery, the portal vein or the wide spread lymph infiltration making a curative resection impossible. In the retrospective analysis of the surgical treatment of 724 patients with GC treated primarily in Europe between 1980 and 1989 no progress has been observed with respect to survival [4]. However, since the last years there are increasing number of studies giving hope for improvement of survival after an aggressive surgical approach in resectable lesions [6-8]. This was especially relevant for tumors infiltrating the liver, the bile duct or the lymphatic nodes. Whereas survival in T1a tumors without infiltration of the muscle layer is generally good and extended CHE (e.g. with wedge resection of the gallbladder bed) is unlikely to improve the outcome in this group, there is still a debate whether T1b tumors (tumor invading through the mucosa into the muscle layer) should be treated like T2 tumors and liver resection combined with lymph dissection of the HL should be performed [9]. In this study there was only one patient with a T1b tumor, but we think that treatment in T1b tumors should not differ from T2 tumors. Thereby we focus mainly on the lymphatic spread of the tumor. It has been shown that lymphatic spread occurs early, before liver involvement and that nodal involvement is a poor prognostic factor [10-14]. However, there were many patients reported in the literature, in whom lymph node metastases had been excised and who survived more than 5 years. Our finding of early involvement of the HL in GC and an incidence of 25% in T2 tumors confirms these reports. Lessons we learned from the course of our patients presenting with recurrent tumors after intended curative resections (see table 2) revealed that treatment strategies in the past focused too much on liver infiltration thereby forgetting the lymphatic spread. In the past, in our department infiltration of the HL has not usually been an indication for lymph dissection or was used as an argument against the feasibility of curative resection. The preliminary report of the German CAE-S/CAMIC registry of CHEs with a postoperative incidental finding of cancer comprises about 245 patients with GC [14]. In the registry there were 118 patients with T2 tumors, 44 patients with T3 tumors and 20 patients with T4 tumors. Altogether, there were only 70 reoperations performed. This demonstrates that an aggressive surgical approach is still not performed everywhere. One major problem in simple CHE is that no definite statement about lymph node infiltration can be done macroscopically and the tumor stage might be underestimated. The missing of lymph node infiltration might result in progressive lymphatic tumor spread especially into coeliacal, peripancreatic and interaortocaval lymphatic nodes leading to rapid and disseminated tumor growth and dismal prognosis. Of importance, there were therefore 70 patients in the CAE-S/CAMIC registry with recurrent disease after a median follow-up of 27 months.

In this study we could demonstrate a better survival of R0-resections in patients after the implementation of a standardized surgical approach for GC ranging from UICC stage Ia (T1b tumors) to stage III (T4 tumors with and without regional lymphatic node metastases). To our opinion the marked difference of the standardized approach in comparison to the individual resections, was the radial dissection of the HL and the resection of the CBD.

Before the implementation of the standard operation, liver resection was usually performed as non-anatomical wedge resection of the gallbladder bed to reach tumor free resection margins. Although this study could not clarify whether anatomic segmentectomy is superior to a non-anatomic wedge resection, we nowadays advocate the anatomical segment IVb/V liver resection with the selective extrahepatic vascular approach. By some authors right sided hemihepatectomy is recommended to respect a security distance of the tumor of about 3 cm. Ogura *et al*. measured the distance between the front of the carcinoma invasion and the resection plane in the hepatic parenchyma [15]. The distance ranged between 12–20 mm after wedge resection, 16–35 mm after resections of segments IV+V and 28–58 mm after extended hepatic resections. By the analysis of the pathological reports of our liver resection specimen we found that a 3 cm distance can also be achieved by 77% of segment IVb/V resections. Moreover we demonstrate in this study that this procedure can usually be done without requiring blood transfusions. Therefore segment IVb/V liver resection is a better alternative than hemihepatectomy as it is sufficient in most cases to achieve a R0 resection and is much less invasive. Only in some cases of T4 tumors hemihepatectomy might be still necessary to completely remove the tumor. Nevertheless, radicality of resection is usually more dependent on lymph dissection than resection of the infiltrated liver tissue or surrounding organs like the small intestine and colon.

Metastatic disease is usually no indication for surgery. In this study we could not demonstrate any difference of solid organ metastases compared to peritoneal tumor seeding in terms of remaining survival time. There is also no benefit of palliative CHE.

Whereas we are convinced of the benefit of the standardized approach, some – primarily Japanese authors – advocate the need of the resection of a "fourths or fifths component", that is the necessity of the routine paraaortal lymph node dissection or even the hepatopancreatoduodenectomy (HPD). Since the pioneer report of HPD by Hanyu *et al*., [16] a large number of patients with UICC stage III GCs was undergoing HPD and five year survival rates from 29% – 87% were reported [17,18]. Despite the evident pathoanatomical rational of this approach, as there is a high incidence of peripancreatic infiltrated lymph nodes, nearly all Western surgeons are hesitant to pursue such an aggressive approach due to its high mortality and morbidity.

Tsukada *et al*. found lymph node metastases in 12% of patients who underwent paraaortic node dissection [3]. However, paraaortic lymphadenectomy provided no survival benefit and led to the same prognosis to that of distant metastases [19]. We did not routinely perform lymph dissection in the interaortocaval compartment. In our series we found every second lymph node biopsy at the celiac trunk positive in T3 tumors and even every biopsy positive in T4 tumors. But in the case of tumor infiltration at the celiac trunk there were usually already metastases detected, thus lymph dissection of the interaortocaval compartment would have rarely been a real curative option.

## Conclusion

The standardized approach is an efficacious treatment for locally advanced gallbladder carcinoma and did improve survival compared to less aggressive surgical resections even in our small cohort. In advanced tumor stages with presence of peripancreatic lymph node disease a more aggressive approach with HPD is limited by its high morbidity. In metastatic disease, palliative surgical procedures should be restricted to bypass measures like the segment III bypass, gastroenterostomy or palliative intestinal resection to ensure quality of life. Our study should encourage other centers to incorporate more aggressive standardized procedures in the treatment of GC. Hopefully, this will enable us to compare multicenter treatment results in the future to better estimate the improvement in the surgical therapy of the usually rare and prognostically serious GC.

## Competing interests

The author(s) declare that they have no competing interests.

## Authors' contributions

**SS **as the first author wrote the manuscript and did the statistical analyses, **ChJ **did the patients follow ups, **TS **was the pathologist and did the histological classifications, **MW **participated in the design of the study and helped with the follow up, **DI **did the assessment of the surgical therapy, **MS **was responsible for the whole study and mostly participated in the design of the study. All authors read and approved the final manuscript.
